# Antimutagenic and Chemopreventive Properties of 6-(Methylsulfinyl) Hexyl Isothiocyanate on TK6 Human Cells by Flow Cytometry

**DOI:** 10.3389/fphar.2020.01242

**Published:** 2020-08-18

**Authors:** Veronica Cocchi, Patrizia Hrelia, Monia Lenzi

**Affiliations:** Department of Pharmacy and Biotechnology, Alma Mater Studiorum University of Bologna, Bologna, Italy

**Keywords:** 6-MITC, antimutagenesis, chemoprevention, micronuclei, flow cytometry, TK6

## Abstract

6-(methylsulfinyl) hexyl isothiocyanate (6-MITC), is the main bioactive compound present in *Wasabia japonica* rhizome. Several scientific studies have shown that 6-MITC possesses interesting antimicrobial, anti-inflammatory, antiplatelet and antioxidant properties which therefore suggested us it could have an interesting chemopreventive potential. In a recent publication, we demonstrated, in two different leukemia cell lines, its ability to modulate several mechanisms supporting its antitumor activity. For this reason, we thought useful to continue the research, by investigating the potential antimutagenic activity of 6-MITC and thus better define its profile as a possible chemopreventive agent. 6-MITC antimutagenic effect against two known mutagenic agents: the clastogen Mitomycin C (MMC) and the aneuplodogen Vinblastine (VINB), was analyzed, in terms of micronuclei frequency decrease, after short- and long- time treatment on TK6 human cells, using a new automated protocol of the “*In Vitro* Mammalian Cell Micronucleous Test” by flow cytometry. The results showed a different behavior of the isothiocyante. In particular, 6-MITC was unable to counteract the MMC genotoxicity, but when it was associated with VINB a statistically significant decrease in the micronuclei frequency was registered. Overall, the results obtained suggest a potential antimutagenic activity of 6-MITC, in particular against the aneuploidogen agents. This ability, to inhibit or counteract the mutations at the cellular level has a great therapeutic value and it represents a mechanism through a chemopreventive agent can express its activity.

## Introduction

6-(Methylsulfinyl) hexil isothiocyanate (6-MITC) is the main bioactive compound present in *Wasabia japonica*, a plant belonging to the *Brassicaceae* family, also called “Japanese radish”. A green paste with a particularly spicy taste is made from the rhizome of this plant, that is used in traditional Japanese cuisine and commonly known as Wasabi (Weil et al., 2004; Weil et al., 2005; Hsuan et al., 2016).

Isothiocyanates have long been considered by the scientific community, for the numerous pharmacological properties demonstrated (Melchini et al., 2013; Lenzi et al., 2014). Several scientific studies have shown that 6-MITC in particular, possesses interesting antimicrobial (Hirokuni et al., 1998; Ko et al., 2016), anti-inflammatory (Uto et al., 2005; Uto et al., 2007; Uto et al., 2012), antiplatelet (Morimitsu et al., 2000) and antioxidant (Mizuno et al., 2011) properties.

These results suggested us a potential interest 6-MITC as a chemopreventive agent. In a recent publication, we therefore demonstrated, in two different leukemia cell lines (Jurkat and HL-60), its ability to modulate several mechanisms supporting its antitumor activity, such as the cyotodifferentiation and apoptosis induction or the cellular proliferation inhibition (Lenzi et al., 2017).

Beside the ability to interact with cellular and molecular targets, crucial in the development of cancer, also the study and the identification of compounds capable of counteracting the genotoxicity, it is recognized of great interest in the field of chemoprevention (Słoczyńska et al., 2010). In fact, if the mutation occurs in a somatic cell it could lead to premature aging, damage to the immune system and promote the development of chronic degenerative diseases, such as cancer (Basu, 2018).

Initially, the mutagenic activity of 6-MITC was evaluated both at short and long times, in order to exclude the mutagenicity of the compound under study, Subsequently, the research continued by analyzing the antimutagen potential of 6-MITC against two known mutagenic agents, characterized by different mechanism of action, *i.e* the clastogen Mitomycin C (MMC) and the aneuploidogen Vinblastine (VINB). For this purpose, we decided to use a new automated protocol of the Micronucleous (MN) Test by flow cytometry (FCM) (Lenzi et al., 2018; Lenzi et al., 2020).

Numerous genotoxicity tests are validated by OECD and some allow to highlight gene mutations, while other permit to show chromosomal aberrations (OECD Overview, 2014-2015). In this work, we select the “*In Vitro* Mammalian Cell Micronucleous Test”, (OECD no. 487, 2016) because the MN represents a sensitive biomarker of both structural chromosomal damages, induced by clastogen agents and numeric chromosomal damages, induced by aneuploidogen agents (OECD Overview 2014-2015, 2017; Lenzi et al., 2020).

Among several cell lines (CHO, V79, CHL/IU, L5178Y and TK6) validated by the OECD guideline no. 487 that can be used to assess the genotoxicity of a xenobiotic, we selected TK6 cells (OECD no. 487, 2016). Our choice is based on the human and not tumorigenic origin of this cell line which better represents the possible effect on human beings. Moreover, since TK6 cells grow in suspension, they are particularly suitable for FCM (Lenzi et al., 2020).

## Materials and Methods

### Reagents

Dimethyl sulfoxide (DMSO), Ethanol, Ethylenediaminetetraacetic acid (EDTA), Fetal Bovine Serum (FBS), L-Glutamine (L-GLU), Mitomycin C (MMC), Nonidet, Penicillin-Streptomycin solution (PS), Potassium Chloride, Potassium Dihydrogen Phosphate, Roswell Park Memorial Institute (RPMI) 1640 medium, Vinblastine (VINB),Water bpc grade, Sodium Chloride, Sodium Hydrogen Phosphate (all purchased from Sigma-Aldrich, St Louis, Missouri, USA), Guava Nexin Reagent, Guava ViaCount Reagent (all purchased from Luminex Corporation, Austin, Texas, USA), RNase A, SYTOX Green, 7-aminoactinomycinD (7-AAD) (all purchased from Thermo Fisher Scientific, Waltham, Massachusetts, USA).

### 6-MITC

6-MITC was purchased from Abcam, Cambridge, UK. The purity of ITC was >98%. The isothiocyanate was dissolved in DMSO up to 97.39mM stock solution and stored in the dark at −20°C. Increasing concentrations of 6-MITC from 0 to 64µM were tested. DMSO concentration was always in the range 0.05–1% in all the experimental conditions.

### Cell Culture

TK6 human lymphoblast cells were purchased by Sigma-Aldrich (St. Louis, Missouri, USA) and were grown at 37°C and 5% CO_2_ in RPMI-1640 supplemented with 10% FBS, 1% L-GLU and 1% PS. To maintain exponential growth, the cultures were divided every third day in fresh medium and the cell density did not exceed the critical value of 9x10^5^ cells/mL.

### Treatments

#### Short-Term Treatment

Aliquot of 2.0x10^5^ of TK6 cells were treated with increasing concentrations of 6-MITC (0 to 64µM) and incubated for 3h followed by 23h of recovery in fresh medium, 26h total, corresponding to two replication cycles, in the presence or absence of MMC (400ng/mL) or VINB (25ng/mL) (co-treatment). We selected these concentrations on the basis of the literature (Sobol et al., 2012) and, as stated in the OECD guideline, we checked that cytotoxicity and cytostasis were lower than 55 ± 5% (OECD no. 487, 2016).

#### Long-Term Treatment

Aliquot of 2.0x10^5^ of TK6 cells were treated with increasing concentrations of 6-MITC (0 to 32µM) and incubated for 26h consecutive, corresponding to two replication cycles, in the presence or absence of MMC (200ng/mL) or VINB (6.25ng/mL) (co-treatment). We selected these concentrations on the basis of the literature (Sobol et al., 2012) and, as stated in the OECD guideline, we checked that cytotoxicity and cytostasis were lower than 55 ± 5% (OECD no. 487, 2016).

### Flow Cytometry

All FCM analysis reported below were performed using a Guava easyCyte 5HT flow cytometer equipped with a class IIIb laser operating at 488 nm (Merck, Darmstadt, Germany).

#### Cytotoxicity and Cytostasis Analysis

In order to detect cytotoxicity and cytostasis the percentage of viable cells was evaluated by the Guava ViaCount Assay. Briefly, cells were stained with Guava ViaCount Reagent (containing Propidium Iodide, PI) and analyzed by Guava ViaCount software.

In particular, to assess the cytotoxicity, the results obtained in the sample treated with different concentrations of 6-MITC were normalized on those obtained in the control cultures.

In parallel, the number of cells seeded at time 0 and that measured at the end of the treatment time, was used to check the correct replication in the control cultures and compare it to that measured in the treated cultures using the relative population doubling (RPD).

$$RPD=\frac{\left(No.\;of\;Population\;doublings\;in\;treated\;cultures\right)}{\left(No.\;of\;Population\;doublings\;in\;control\;cultures\right)}x100$$

#### Apoptosis Analysis

The percentage of apoptotic cells was evaluated by the Guava Nexin Assay. Briefly, the percentage of live, apoptotic and necrotic cells was assessed using the Guava Nexin Reagent (containing 7-AAD and Annexin-V-PE) and analyzed by Guava Nexin software.

The results obtained were expressed as apoptotic fold increase of treated cultures compared to control cultures and were used to select MNs test concentrations. In fact, it is necessary that the percentage of apoptotic cells measured in treated cultures is comparable to that present in the control cultures, in order to avoid possible false positives, due to the presence of apoptotic bodies.

#### Genotoxicity Analysis

**Figure 1:**
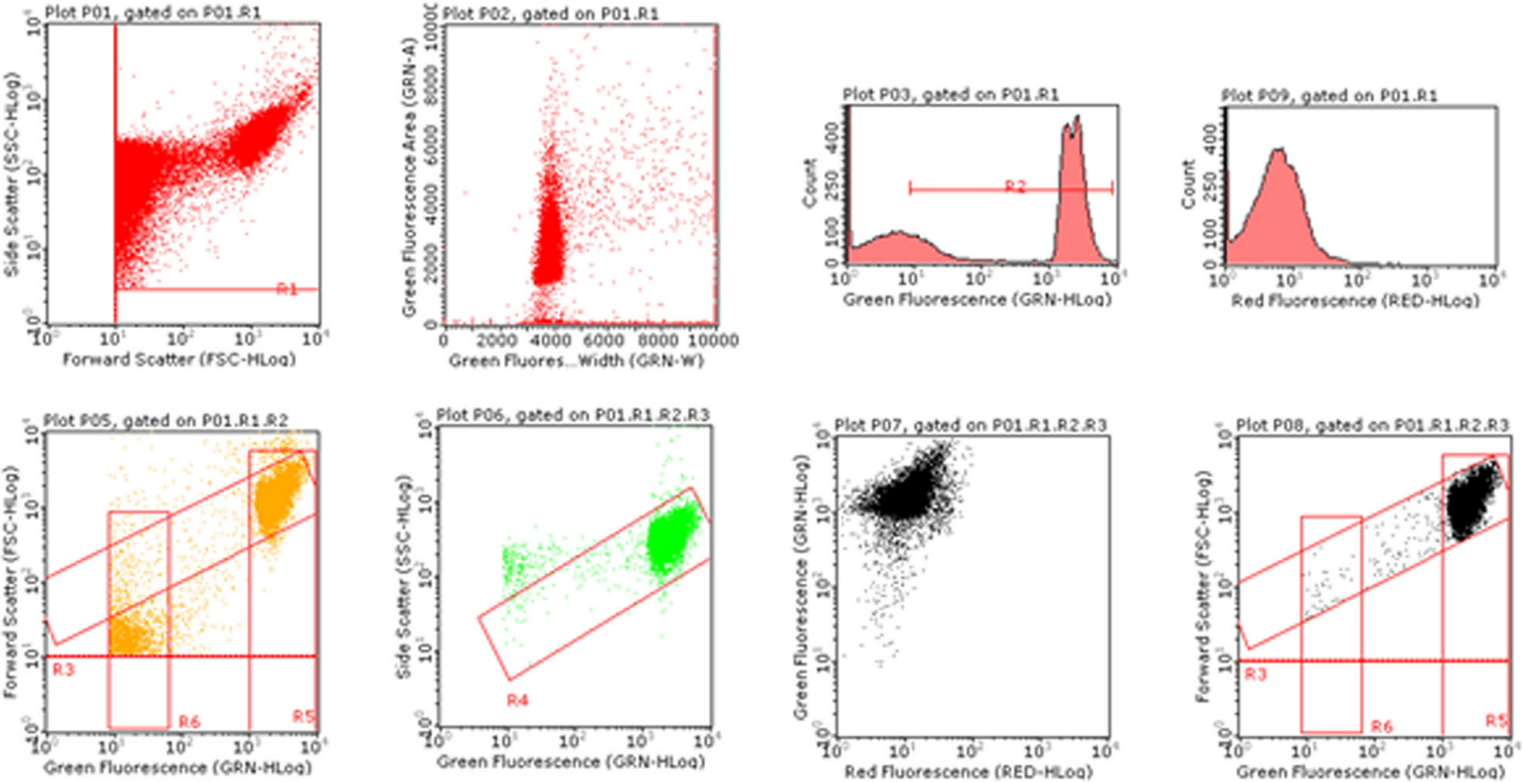
Image of bivariate plots of nuclei and MNs analyzed by Guava Incyte software.

The analysis of the MNs frequency was performed using an automated protocol by Lenzi et al. (2018). Briefly, after 3 or 26h from 6-MITC exposure, aliquots of 7x10^5^ cells were collected and stained with 7-AAD and SYTOX Green. The discrimination between nuclei and MNs was performed on the basis of the different size analyzed by Forward Scatter (FSC), and the different intensity of green fluorescence. For each sample the MNs frequency was measured on 10,000 nuclei derived from viable and proliferating cells on the basis of different red fluorescence. The results were expressed as MNs frequency fold increase in treated cultures compared to that present in the control cultures (**Figure 1**).

### Statistical Analysis

All results were expressed as mean ± SEM of at least five independent experiments. For the statistical analysis of the viability, apoptosis and MNs we used the Analysis of Variance for paired data (repeated ANOVA), followed by Dunnett or Bonferroni as post-test. All the statistical analyses were performed using Prism Software 4.

## Results

### Mutagenesis of 6-MITC

#### Short-Term Treatment (3h+23h)

In order to select the concentrations to be used in the following experiments, we, first, evaluated the cytotoxic and cytostatic potential of 6-MITC after 3h treatment followed by 23h of recovery in complete medium at different concentrations (0, 2, 4, 8, 16, 32, and 64µM). In fact, the OECD guideline no. 487 recommends proceeding to assess the genotoxicity of a xenobiotic, only if the highest concentration tested shows cytotoxicity and cytostasis at most equal to 55 ± 5% (OECD no. 487, 2016).

**Figure 2:**
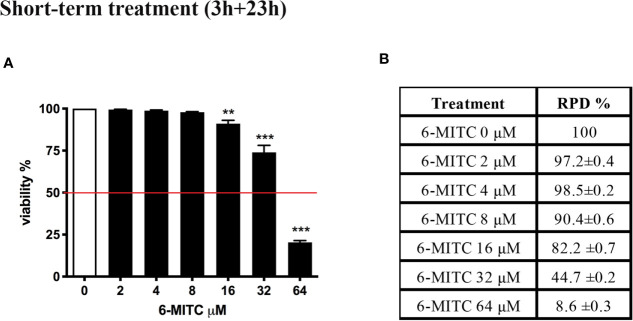
Effect of 6-MITC on cytotoxicity and cytostasis after short term treatment. Percentage of viability **(A)** and RPD **(B)** in TK6 cells treated with 6-MITC for 3h followed by 23h of recovery in complete medium. Each bar represents the mean ± SEM of five independent experiments. Data were analysed using repeated ANOVA followed by Dunnet post-test. **p < 0.01 *vs* 0; ***p < 0.001 *vs* 0.

**Figure 2** shows that the viability remains abundantly higher than the threshold (red line) required by the OECD guideline at all concentrations tested, except for the 6-MITC 64µM (**Figure 2A**).

At the same time, using RPD value, the cytostasis was checked. Similarly, to cytotoxicity, all concentrations tested, except the 6-MITC 64µM, respect the threshold (**Figure 2B**).

**Figure 3:**
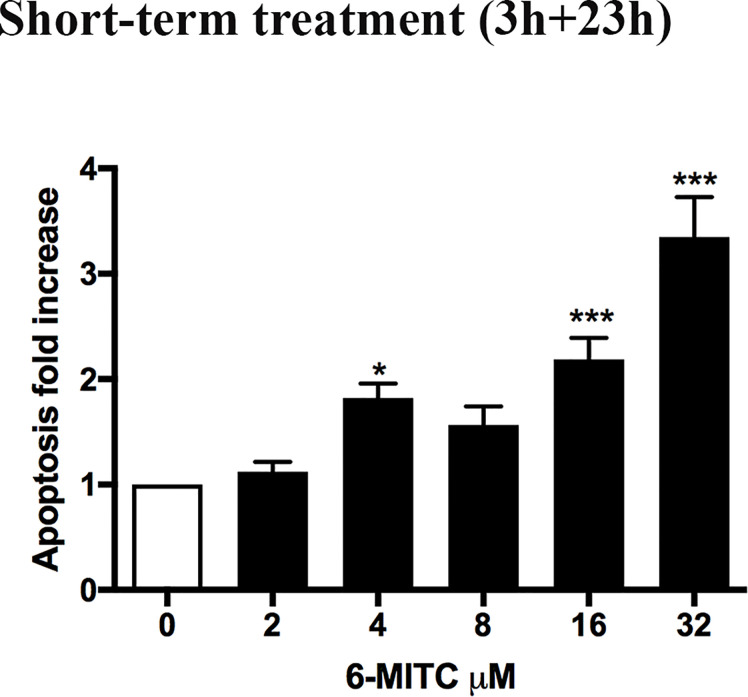
Effect of 6-MITC on apoptosis after short term treatment. Apoptosis fold increase in TK6 cells treated with 6-MITC for 3h followed by 23h of recovery in complete medium. Each bar represents the mean ± SEM of five independent experiments. Data were analysed using repeated ANOVA followed by Dunnet post-test. *p < 0.05 *vs* 0; ***p < 0.001 *vs* 0.

Subsequently, the induction of apoptosis was evaluated as an alternative cell death mechanism, in order to avoid the possible confounding effect of apoptotic bodies with MNs. In particular, with respect to the control cultures, a similar apoptotic trend was detected a 2, 4 and 8μM, while a two and three-time increase was detected at 16 and 32μM, respectively (**Figure 3**).

Therefore, on the basis of the obtained results, 2 and 4µM concentrations were selected to be used to assess the potential genotoxicity induced by 6-MITC.

**Figure 4:**
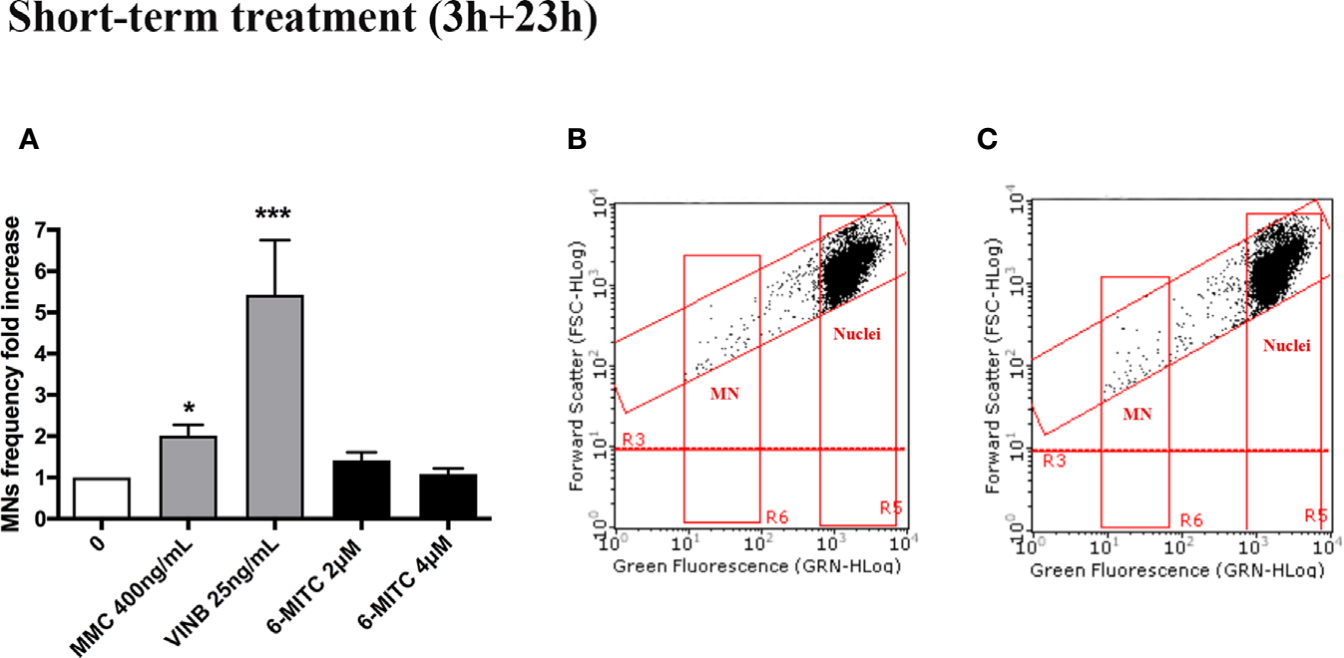
Effect of 6-MITC on mutagenesis after short term treatment. MNs frequency fold increase **(A)** and dot plot obtained by FCM in the control cultures **(B)** and 6-MITC 4µM treated cultures **(C)** on TK6 cells after 3h treatment followed by 23h of recovery in complete medium. Each bar represents the mean ± SEM of five independent experiments. Data were analysed using repeated ANOVA followed by Bonferroni post-test. *p < 0.05 *vs* 0; ***P < 0.01 *vs* 0.

For this purpose, the MNs frequency was measured in control and treated cultures and compared with MMC 400ng/mL and VINB 25ng/mL, used as a positive control. As shown in **Figure 4** the MNs frequency increase registered in 6-MITC treated cultures was not statistically significant compared to the control cultures, while an increase equal to two and five time was detected in the MMC and VINB treated culture, respectively (**Figures 4A–C**).

#### Long-Term Treatment (26h)

In order to completely exclude the genotoxicity of a substance, the OECD guideline no. 487 suggests to check the effect also after a long-term treatment (OECD no. 487, 2016). For this reason, TK6 cells were treated with different concentrations of 6-MITC (0, 1, 2, 4, 8, 16μM) for 26h.

Similarly, to what described above for the short-time treatment, also in this case, initially were selected non-cytotoxic and non-cytostatic concentrations.

**Figure 5:**
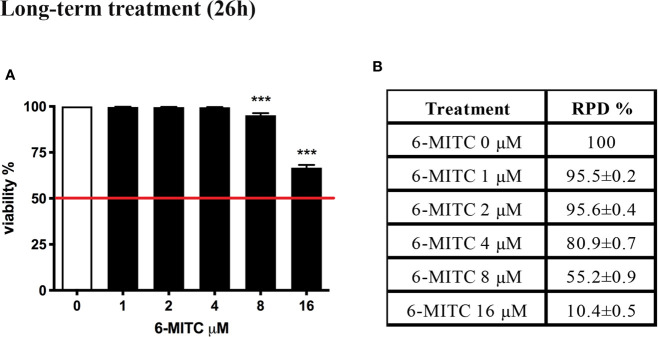
Effect of 6-MITC on cytotocicity and cytostasis after long term treatment. Percentage of viability **(A)** and RPD **(B)** in TK6 cells treated with 6-MITC for 26h. Each bar represents the mean ± SEM of five independent experiments. Data were analysed using repeated ANOVA followed by Dunnet post-test. *** p < 0.001 *vs* 0.

**Figure 5** shows that the viability remains abundantly higher than the 50% (red line) for all concentrations tested (**Figure 5A**), while the RPD values respect the threshold at all concentrations tested, except the 16μM. In this case a cytostasis equal to 89.6% was observed and so a cell proliferation equal to 10.4% (**Figure 5B**). For this reason, the 16μM concentration was excluded from the apoptosis test.

**Figure 6:**
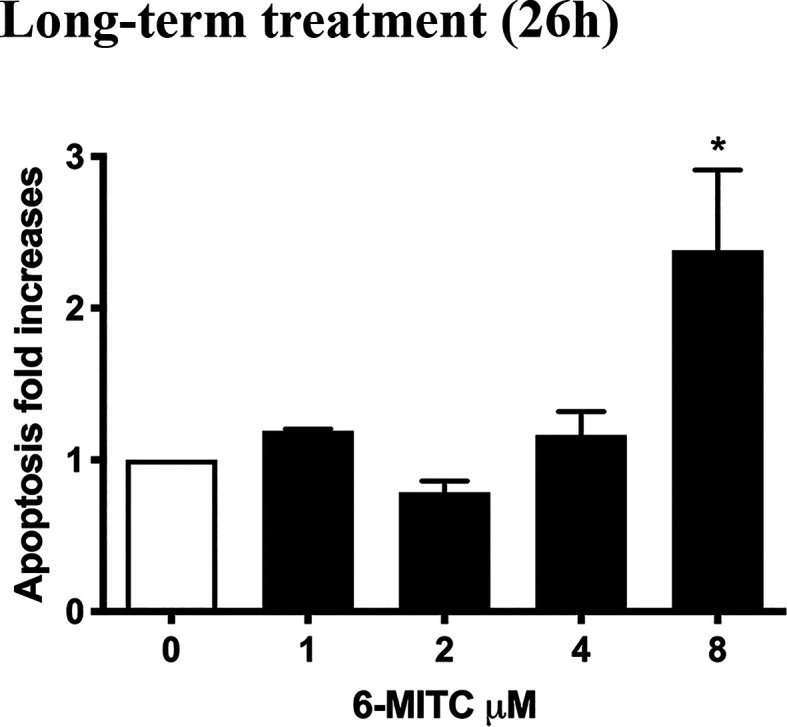
Effect of 6-MITC on apoptosis after long term treatment. Apoptosis fold increase in TK6 cells treated with 6-MITC for 26h. Each bar represents the mean ± SEM of five independent experiments. Data were analysed using repeated ANOVA followed by Bonferroni post-test. *p < 0.05 *vs* 0.

Annexin V-PE/7-AAD double staining allowed to calculate the percentage of apoptotic cells. As shown in **Figure 6** only for the 6-MITC 8µM, compared to the control cultures, a population doubling was detected.

Therefore, on the basis of the obtained results, 1 and 2µM concentrations were selected to be used to assess the potential genotoxicity induced by 6-MITC.

**Figure 7:**
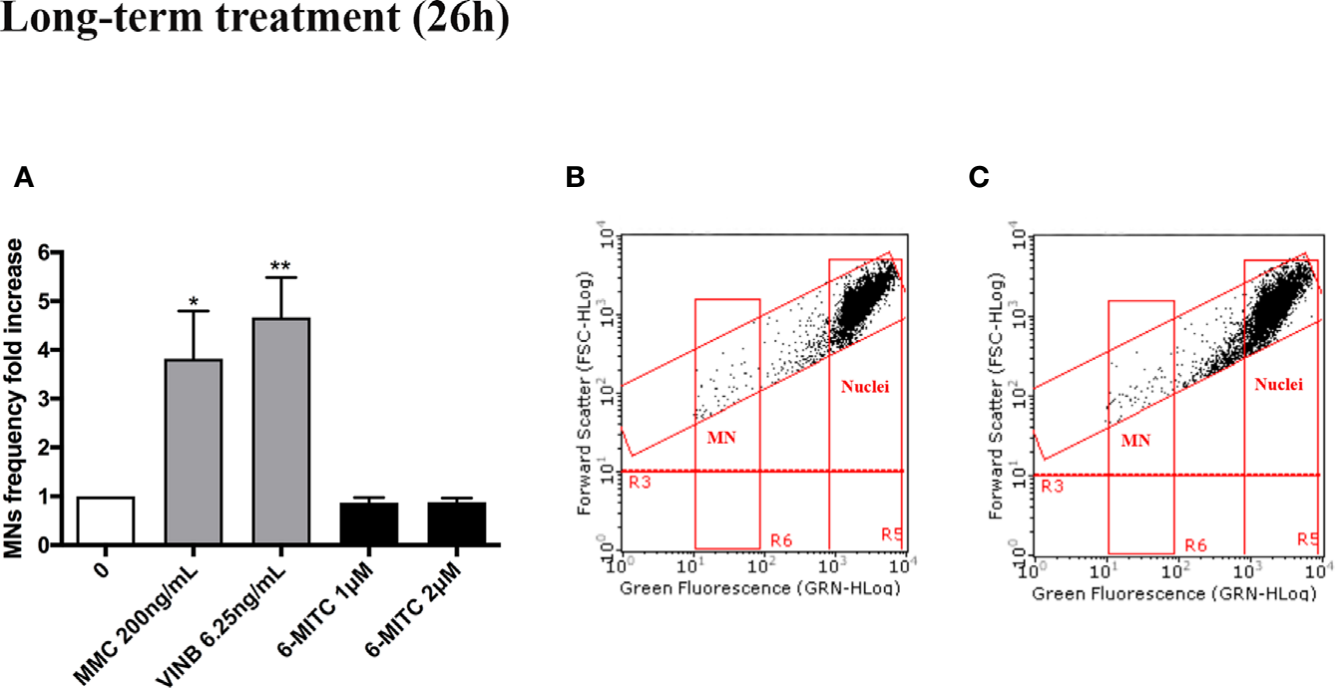
Effect of 6-MITC on mutagenesis after long term treatment. MNs frequency fold increase **(A)** and dot plot obtained by FCM in the control cultures **(B)** and 6-MITC 2 µM treatment cultures **(C)** on TK6 cells after 26h of treatment. Each bar represents the mean ± SEM of five independent experiments. Data were analysed using repeated ANOVA followed by Bonferroni post-test. *p < 0.05 *vs* 0; **P < 0.01 *vs* 0.

As shown in **Figure 7** also in this case, analogously to the short-term treatment, 6-MITC not induced mutagenic activity. In fact, a MNs frequency statistically significant increase, was not registered in all 6-MITC treated cultures (compared to the control cultures), while a four- and five- time increase was detected for the mutagens MMC 200ng/ml and VINB 6.25ng/ml, respectively (**Figures 7A–C**).

### Antimutagenesis of 6-MITC

#### Short-Term Treatment (3h+23h)

Once the non-mutagenicity of the isothiocyanate was demonstrated to both treatment conditions, the study continued evaluating the possible 6-MITC antimutagenic activity, against the known mutagens previously used as positive control (MMC and VINB), similarly after short- and long- term treatment.

**Figure 8:**
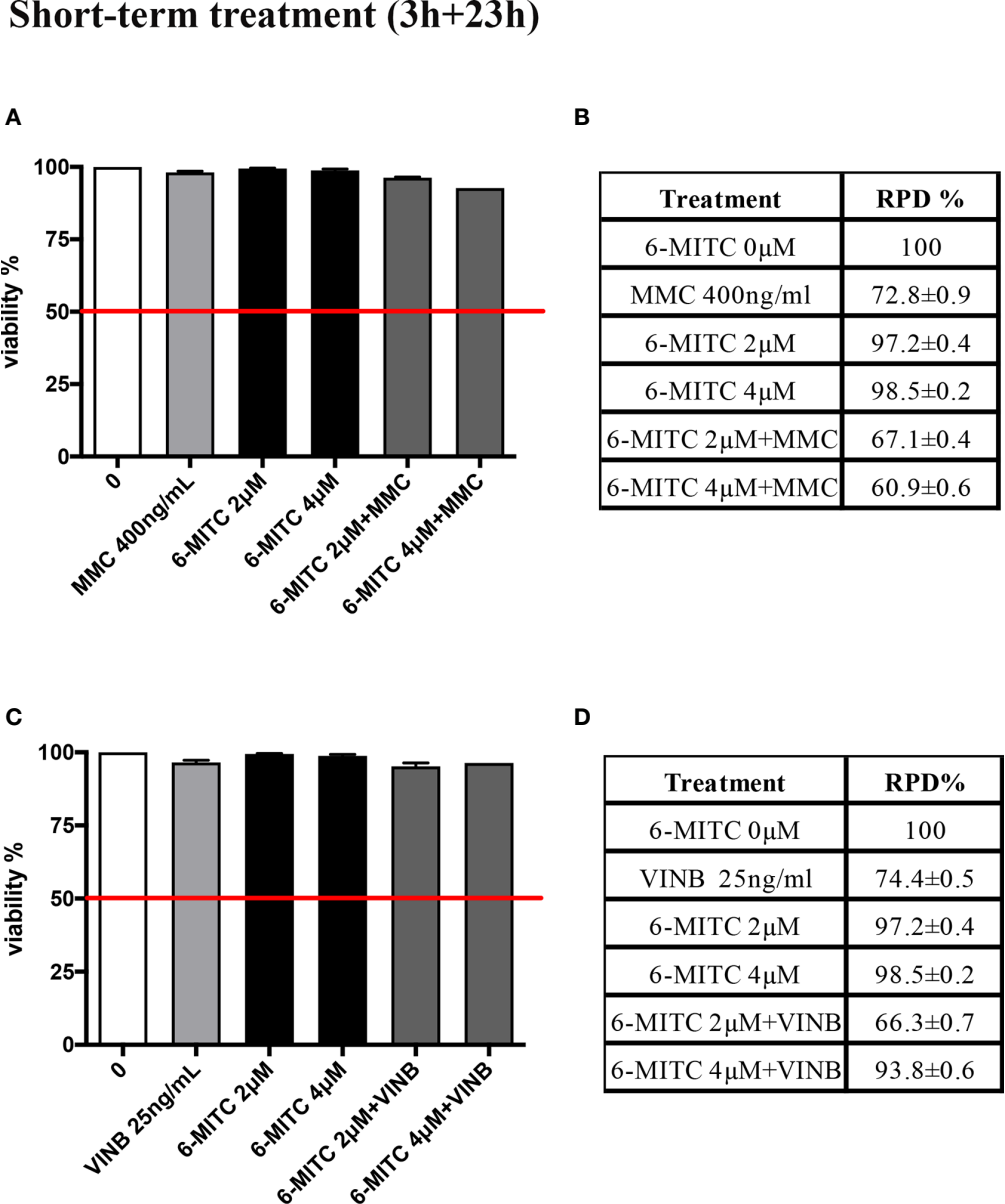
Effect of 6-MITC on cytotoxicity and cytostasis after short term treatment Percentage of viability in TK6 cells treated with 6-MITC for 3h followed by 23h of recovery in complete medium in presence or absence of MMC 400ng/mL **(A)** or VINB 25ng/mL **(C)** and relative RPD values for MMC 400ng/mL **(B)** or VINB 25ng/mL **(D)**. Data represents the mean ± SEM of five independent experiments. Data were analysed using repeated ANOVA followed by Dunnet post-test.

A co-treatment of 3h, followed by 23h of recovery in complete medium, was performed and, also in this case, the cytotoxicity, cytostasis and apoptosis were checked, before proceeding with the genotoxicity analysis. As show in **Figure 8** cell viability (**Figures 8A, C**) and RPD value (**Figures 8B, D**) were abundantly above the threshold established by the OECD guideline no. 487 (**Figures 8A–D**).

**Figure 9:**
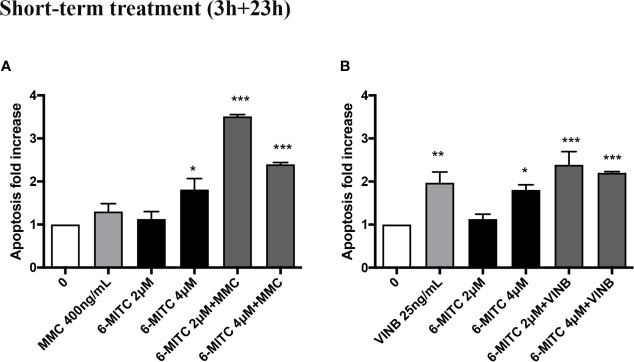
Effect of 6-MITC on apoptosis after short term treatment. Apoptosis fold increase in TK6 cells treated with 6-MITC for 3h followed by 23h of recovery in complete medium in presence or absence of MMC 400ng/mL **(A)** or VINB 25ng/mL **(B)**. Each bar represents the mean ± SEM of five independent experiments. Data were analysed using repeated ANOVA followed by Dunnet post-test. *p < 0.05 *vs* 0; **p < 0.01 *vs* 0; ***p < 0.001 *vs* 0.

An average apoptosis fold increase equal to three time in MMC+6-MITC associations treated cultures respect to the control cultures was observed (**Figure 9A**), while in VINB+6-MITC associations treated cultures an increase on average equal to two times respect to the control cultures was measured (**Figure 9B**).

Overall, the results obtained allowed to proceed with the MN test and to demonstrate the 6-MITC ability to counteract only the VINB mutagenic effect but not the MMC DNA-damage.

**Figure 10:**
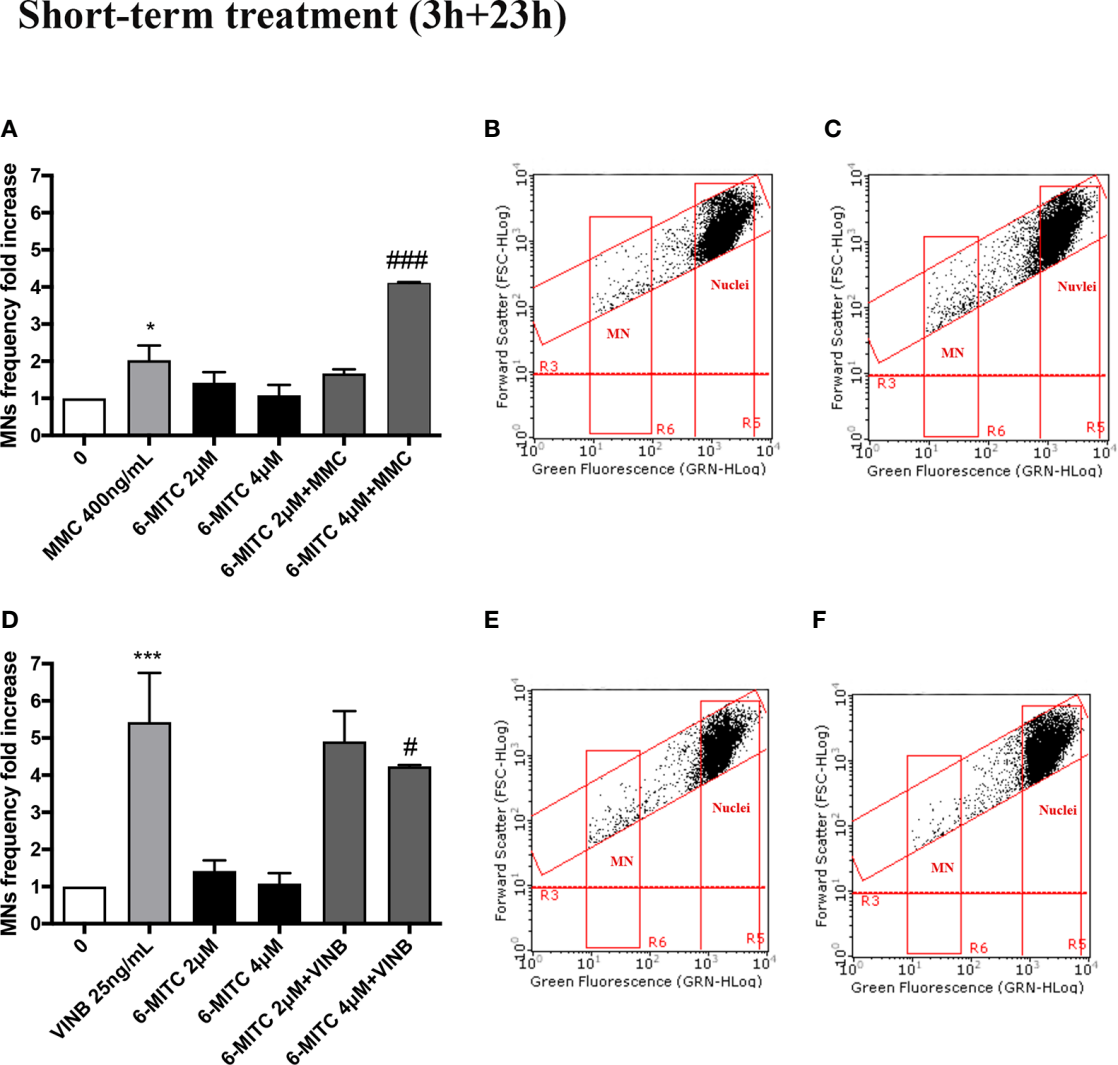
Effect of 6-MITC on antimutagenesis after short term treatment. MNs frequency fold increase on TK6 cells treated for 3h with 6-MITC, followed by 23h of recovery in complete medium, in presence or absence of MMC 400ng/mL **(A)** or VINB 25ng/mL **(D)**. Dot plot obtained by FCM of MMC 400ng/mL **(B)**, MMC400ng/mL + 6-MITC 4µM **(C)**, VINB 25ng/mL **(E)** and VINB 25ng/mL + 6-MITC 4µM **(F)**. Each bar represents the mean ± SEM of five independent experiments. Data were analysed using repeated ANOVA followed by Dunnet or Bonfferoni post-test. *p < 0.05 *vs* 0; ***P < 0.001 *vs* 0. ^###^p < 0.001 *vs* MMC; ^#^p < 0.05 *vs* VINB.

In particular, the MNs frequency increase in the MMC treated cultures in presence of 6-MITC 2µM was comparable than cultures treated with the only mutagen MMC, while the co-treatment MMC and 6-MITC 4µM shown a MNs frequency statistically significant increase (4.1 times *vs* 2.0 times in MMC) (**Figures 10A–C**). On the contrary, in the case of aneuploidogen VINB, a MNs frequency decrease was observed for both 6-MITC associations tested with respect to cultures treated with the mutagen alone, which reaches statistical significance at the highest concentration tested (5.4 times *vs* 4.2 times) (**Figures 10D–F**).

#### Long-Term Treatment (26h)

**Figure 11:**
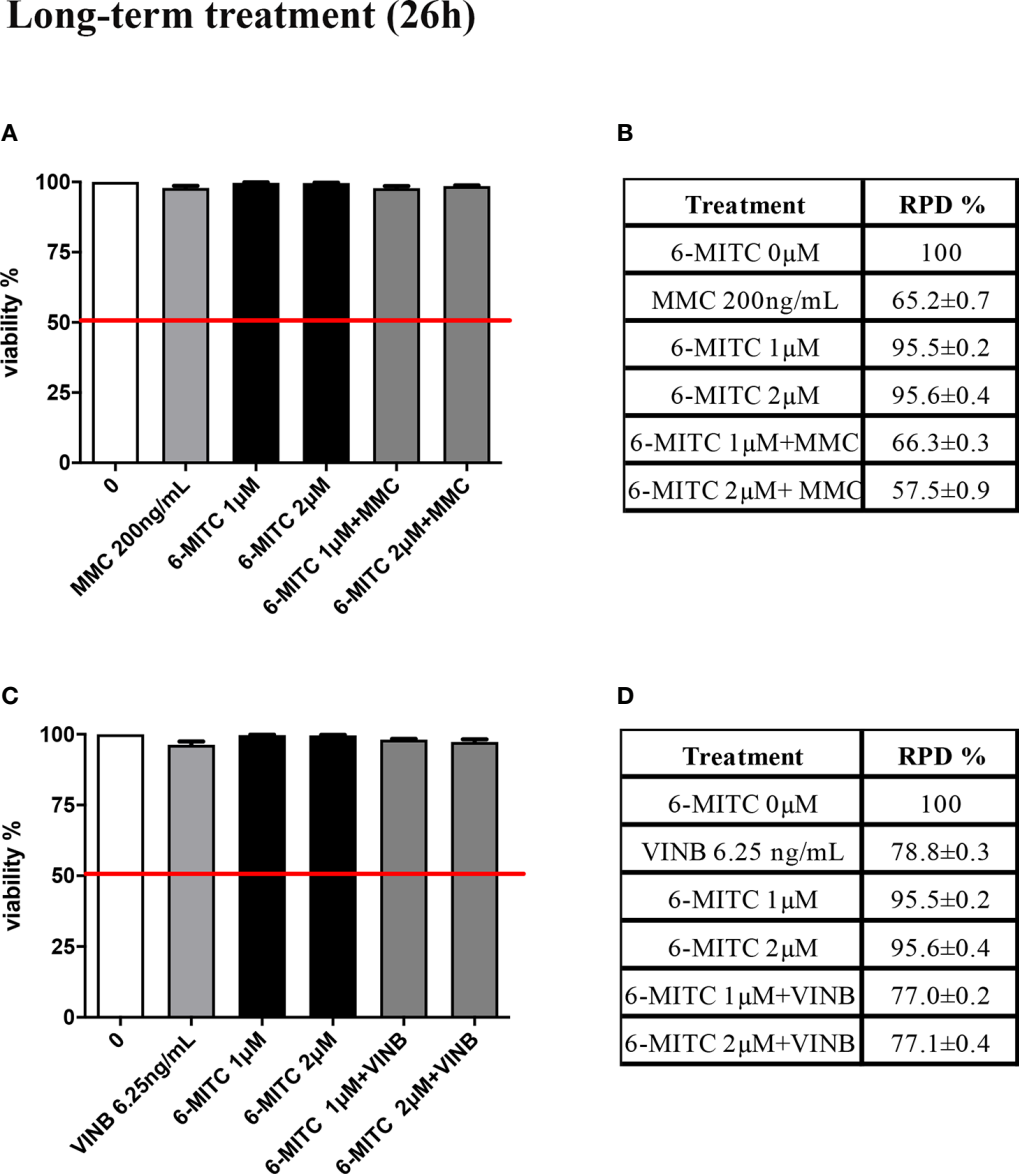
Effect of 6-MITC on cytotocicity and cytostasis after long term treatment. Percentage of viable in TK6 cells treated with 6-MITC for 26h in presence or absence of MMC 200ng/mL **(A)** or VINB 6.25ng/mL **(C)** and relative RPD values for MMC 200ng/mL **(B)** or VINB 6.25ng/mL **(D)**. Each bar represents the mean ± SEM of five independent experiments. Data represents the mean ± SEM of five independent experiments Data were analysed using repeated ANOVA followed by Dunnet post-test.

The study was concluded by evaluating the antimutagenic activity of 6-MITC at 26h. Similarly, to the short-term treatment, cytotoxicity and cytostasis values respected the established threshold at all the conditions analyzed (**Figures 11A–D**).

**Figure 12:**
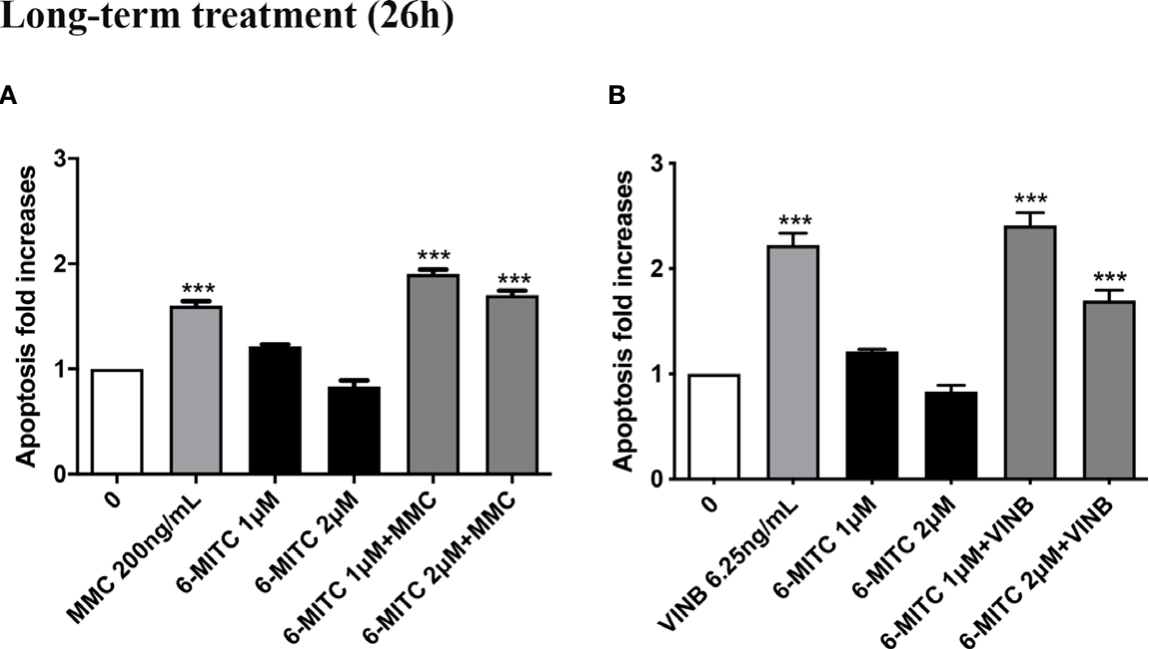
Effect of 6-MITC on apoptosis after long term treatment. Apoptosis fold increase in TK6 cells treated with 6-MITC for 26h in presence or absence of MMC 200ng/mL **(A)** or VINB 6.25ng/mL **(B)**. Each bar represents the mean ± SEM of five independent experiments. Data were analysed using repeated ANOVA followed by Dunnet post-test. ***p < 0.001 vs 0.

Moreover, **Figure 12** show that the apoptosis fold increase reached a doubling in the cultures treated whit VINB alone and in presence of 6-MITC 1µM (**Figures 12A, B**).

**Figure 13:**
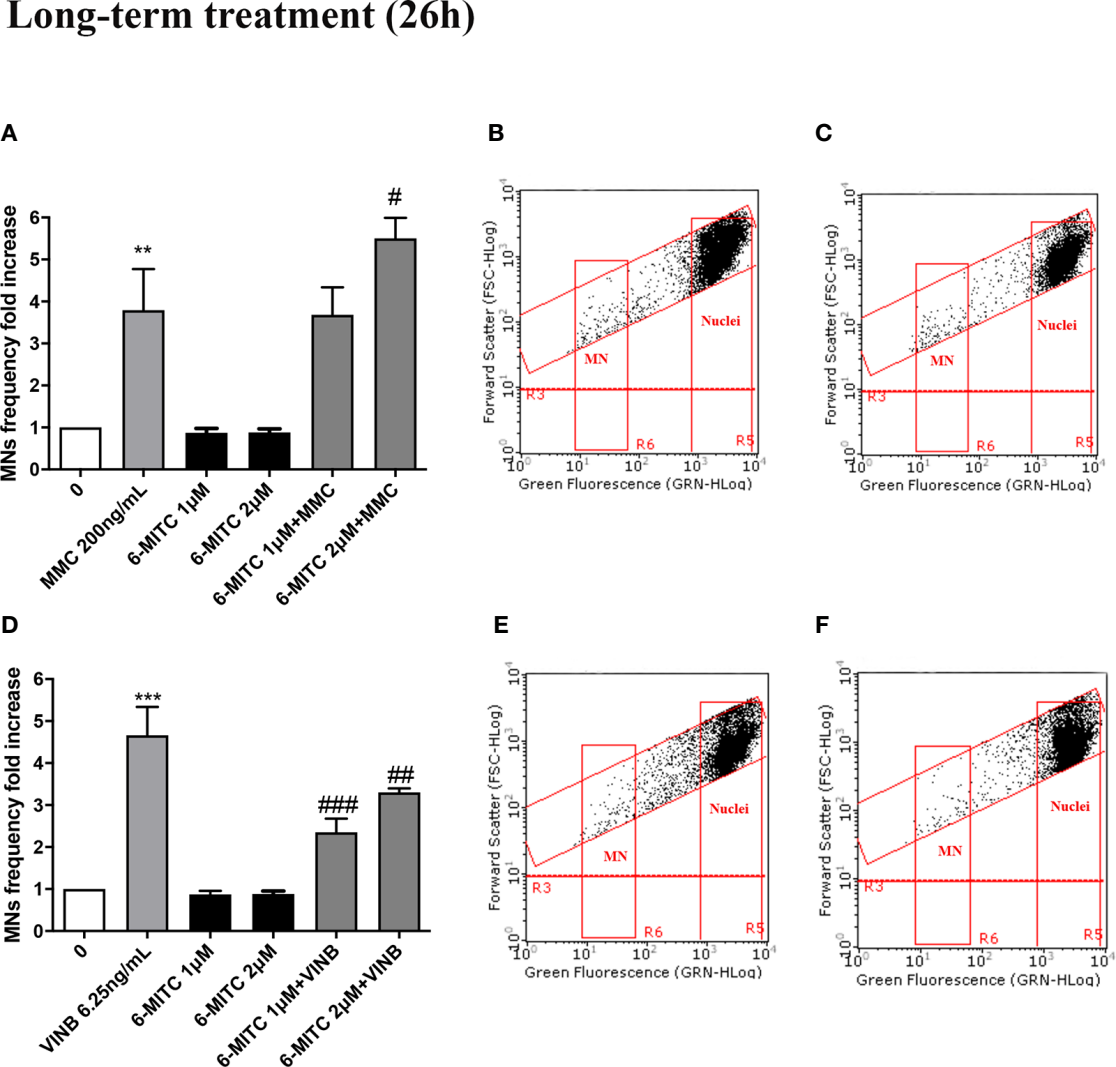
Effect of 6-MITC on antimutagenesis after long term treatment. MNs frequency fold increase on TK6 cells treated for 26h with 6-MITC in presence or absence of MMC 200ng/mL **(A)** or VINB 6.25ng/mL **(D)**. Dot plot obtained by FCM of MMC 200ng/mL **(B)**, MMC 200ng/mL + 6-MITC 2µM **(C)**, VINB 6.25ng/mL **(E)** and VINB 6.25ng/mL + 6-MITC 2µM **(F)**. Each bar represents the mean ± SEM of five independent experiments. Data were analysed using repeated ANOVA followed by Dunnet or Bonfferoni post-test. **p < 0.01 *vs* 0; ***P < 0.001 *vs* 0. ^#^p < 0.05 *vs* MMC; ^##^p < 0.01 *vs* VINB; ^###^p < 0.001 *vs* VINB.

Therefore, checked cytotoxicity, cytostasis and apoptosis, the study ended by evaluating the 6-MITC antimutagen activity, after 26h treatment. The MN test confirmed the results obtained at the short term treatment. Infact, also in this case, the association with MMC led to a statistically significant increase in MNs frequency at the highest concentration tested, compared to the treatment with the clastogen alone (3.8 times *vs* 5.5 times) (**Figures 13A–C**) whereas, the association with the VINB reduced in a statistically significant manner the MNs frequency respet to the treatment with aneuploidogen alone at both concentrations tested (2.3 times and 3.3 times *vs* 4.7 times) (**Figures 13D–F**).

## Discussion

According to our knowledge, no study has addressed the antigenotoxicity of 6-MITC, the main bioactive compound present on *W. japonica*, and very little information are available concerning the whole extract of this plant. In fact, bibliographic research, conducted on the main databases (*i.e.* PubMed from MEDLINE and Scopus from Elsevier) allowed us to identify only two publications. In particular, Kinae and collaborators demonstrated, using the Ames Test, the antimutagenic activity (in terms of gene mutations) of wasabi root, against the 2-amino-3,8-dimethylimidazo[4,5-f]quinoxaline, a well-known mutagen/carcinogen compound present in broiled fish and meat (Kinae et al., 2000).

More recently, the study conducted by Shimamura et al. documented, through Micronucleus Test and Alkaline Comet Assay, the inhibitory effect of Japanese horseradish, on the acrylamide formation and genotoxicity (Shimamura et al., 2017).

These evidences suggest us to verify if the proven *W. japonica* antimutagenic activity was attributable to the 6-MITC.

Despite Wasabi has long been used in traditional Japanese cuisine, it was initially checked the absence of 6-MITC mutagenicity. For this purpose, the non-cytotoxic and cytostatic doses, after short- and long- term treatment of TK6 cells, were defined. In fact, the OECD guideline no.487 recommends proceeding with the evaluation on genotoxicity, only if the treated population shows a viability and cell proliferation of at least 40% when compared to the control cultures (OECD no. 487, 2016).

At the same time, the induction of apoptosis was analyzed, as cell death alternative mechanism and in order to exclude false positive results, due to the possible confounding between apoptotic bodies and MNs by FCM. Overall, based on the results obtained, the concentrations to be used for the evaluation of mutagenicity were selected and, as can be easily predictable, 6-MITC did not show any mutagenic activity both after 3 and 26h treatment.

Subsequently, the study focused on the analysis of the isothiocyanate antimutagenic potential, against two known mutagenic agents: the clastogen MMC and the aneuploidogen VINB.

MMC is characterized by a complex mechanism of action, being able to generate monoalkylation or dialkylation products, and to form covalent cross-linking, between the DNA complementary strands. This interaction prevents strands separation, inhibits DNA replication and causes its break (Tomasz, 1995). Furthermore, MMC generates radical oxygen species such as O_2_, H_2_O_2_, OH*, so the association with antioxidant molecules represent a possible approach to prevent DNA damage (Garcia et al., 2006; Unal et al., 2013). Since the antioxidant properties of wasabi have long been demonstrated (Morimitsu et al., 2002; Lee et al., 2010), it made sense to hypothesize that it was able to counteract the MMC genotoxicity. However, in the present research not only a protective effect was not observed, but even, when 6-MITC is associated with MMC, a statistically significant increase in the MNs frequency was registered. At the moment, exclusively on the basis of the results obtained, it’s difficult to hypothesize a possible explanation of this increase. Certainly, the data must be checked on a greater number of mutagens, to verify if it is common to all clastogen agents or if it is peculiar of MMC.

On the contrary, the isothiocyanate has shown to counteract the mutagenic capacity of the aneuploidogen VINB, which acts at the level of cellular mitosis, by preventing tubulin polymerization and consequently, inhibiting the microtubules aggregation (Navarro et al., 1989).

The statistical analysis evidenced a significant decrease in the MNs frequency equal to about on half after the long treatment with 6 MITC 1µM concentration.

Overall, our work suggests to impute to 6-MITC an antimutagenic capacity. Our findings, are preliminary, since they are obtained against only two mutagens, but allow to highlight the possible mechanism underlying this activity.

In fact, from our data it seems that the isothiocyanate does not counteract the structural DNA damage, but rather the genomic DNA damage, highlighting the possibility that it acts on the mitotic spindle formation or at the chromosomal segregation time.

Alternatively, the co-treatment could suggest a direct extracellular interaction between the isothiacyanate and the mutagenic agent.

These hypothesis needs to be confirmed on a greater number of mutagens, but from the present research emerges an additional interesting biological potential of the 6-MITC. Indeed, the ability to inhibit or counteract the mutations at the cellular level has a great therapeutic value and it represents a less investigated mechanisms through which a chemopreventive agent can express its activity (Amkiss et al., 2013; Cristóbal-Luna et al., 2018). In conclusion, our work, in addition to the induction of apoptosis and the inhibition of cellular proliferation, previously demonstrated (Lenzi et al., 2017), better defines the chemopreventive profile of this interesting isothiocyanate.

## Data Availability Statement

The datasets generated for this study are available on request to the corresponding author.

## Author Contributions

PH and ML designed the project and supervised the experiments. VC performed the experiments and data analysis. VC writing—original draft preparation. VC, PH, and ML writing—review and editing. All authors contributed to the article and approved the submitted version.

## Conflict of Interest

The authors declare that the research was conducted in the absence of any commercial or financial relationships that could be construed as a potential conflict of interest.
